# Spinal Shortening for Recurrent Tethered Cord Syndrome via a Lateral Retropleural Approach: A Novel Operative Technique

**DOI:** 10.7759/cureus.1632

**Published:** 2017-08-31

**Authors:** Jeffrey A Steinberg, Arvin R Wali, Joel Martin, David R Santiago-Dieppa, David Gonda, William Taylor

**Affiliations:** 1 Department of Neurosurgery, University of California, San Diego

**Keywords:** tethered cord syndrome, minimally invasive, lateral, spine-shortening, vertebral osteotomy

## Abstract

Spine shortening via vertebral osteotomy (SSVO) for recurrent tethered cord syndrome (TCS) is a novel surgical technique that avoids the complication profile associated with revision detethering. While SSVO has previously been described via a posterior approach, we describe a lateral retropleural approach for SSVO in recurrent TCS in a 21-year-old female.

Our patient presented with progressive lower extremity weakness, bowel and bladder incontinence, and back pain in the setting of childhood repair of myelomeningocele and two previous detethering procedures. SSVO was offered to the patient as further detethering was deemed to have significant risk. A discectomy at T11-T12 via the lateral retropleural approach was performed, followed by a T12 partial corpectomy removing the vertebral body down to the inferior aspect of the T12 pedicle, followed by the removal of the ipsilateral pedicle. The T10, T11, L1, and L2 pedicle screws were then placed in the prone position and temporary rods were placed for temporary stability, followed by a laminectomy at T12 and a facetectomy for posterior element release. The remaining pedicle was removed, permanent rods were sequentially placed, and spinal column shortening was achieved by compression against the rods. Standing lateral radiographs demonstrated 19 millimeters (mm) of shortening after the intervention. The patient remained at her neurologic baseline postoperatively. At the six-month follow-up, the patient reported decreased lower extremity radicular pain and improved bowel and bladder function.

This operative report demonstrates that SSVO via a lateral retropleural approach is a viable treatment for the recurrence of TCS. The advantages of this minimally invasive approach compared to the posterior approach are direct access to the vertebral body and disc space, avoiding the need to operate around the spinal cord. Further studies are necessary to assess this minimally invasive approach to spinal shortening and to see if a complete minimally invasive approach is possible.

## Introduction

Tethered cord syndrome (TCS) is a progressive neurologic condition due to vertical traction and the stretching of the spinal cord, causing lower back pain, leg pain, lumbosacral weakness, lower extremity numbness, and fecal/urinary incontinence [1-2]. A thickened filum terminale or other congenital lesions, such as spinal lipomas, diastematomyelia, myelomeningocele, and spina bifida, are associated with TCS [2-3]. Correction of these congenital spinal lesions can result in post-surgical scarring, which subsequently leads to TCS in 10%-30% of patients [4-5]. Patients presenting with TCS typically undergo direct spinal cord detethering as first-line management, often as young children or infants. Prompt intervention after diagnosis is associated with improved clinical outcomes [6-7] with the first-time detethering improving 50% of the patients at the 20-year follow-up. The surgical risks of detethering include a cerebrospinal fluid (CSF) leak in 15% of the patients and neurologic injury in three percent of the patients [3].

Retethering of the spinal cord is a common occurrence as fibrous tissue develops, causing a recurrence of symptoms in as many as 25% of the patients [8-10]. The management of the recurrent tethered cord syndrome is significantly more challenging because of scar tissue and adhesions to the spinal cord, dura, and spinal nerve roots. Revision detethering is associated with increased operative morbidity with a subsequent recurrence of TCS, resulting in a progressive neurologic decline in 5%-50% of patients [11-14].

A relatively novel and alternative surgical intervention for the recurrence of TCS involves spine-shortening vertebral osteotomy (SSVO) [12,15]. This procedure indirectly reduces traction on the spinal cord by shortening the length of the spinal column. Reports of this procedure describe a posterior approach with comparable outcomes to successful redo detethering while significantly reducing the complications of CSF leak and direct spinal cord injury [16-17]. In this operative case report, we describe the first lateral approach to SSVO for recurrent TCS in a patient with a history of spina bifida.

## Technical report

Case presentation

**Figure 1:**
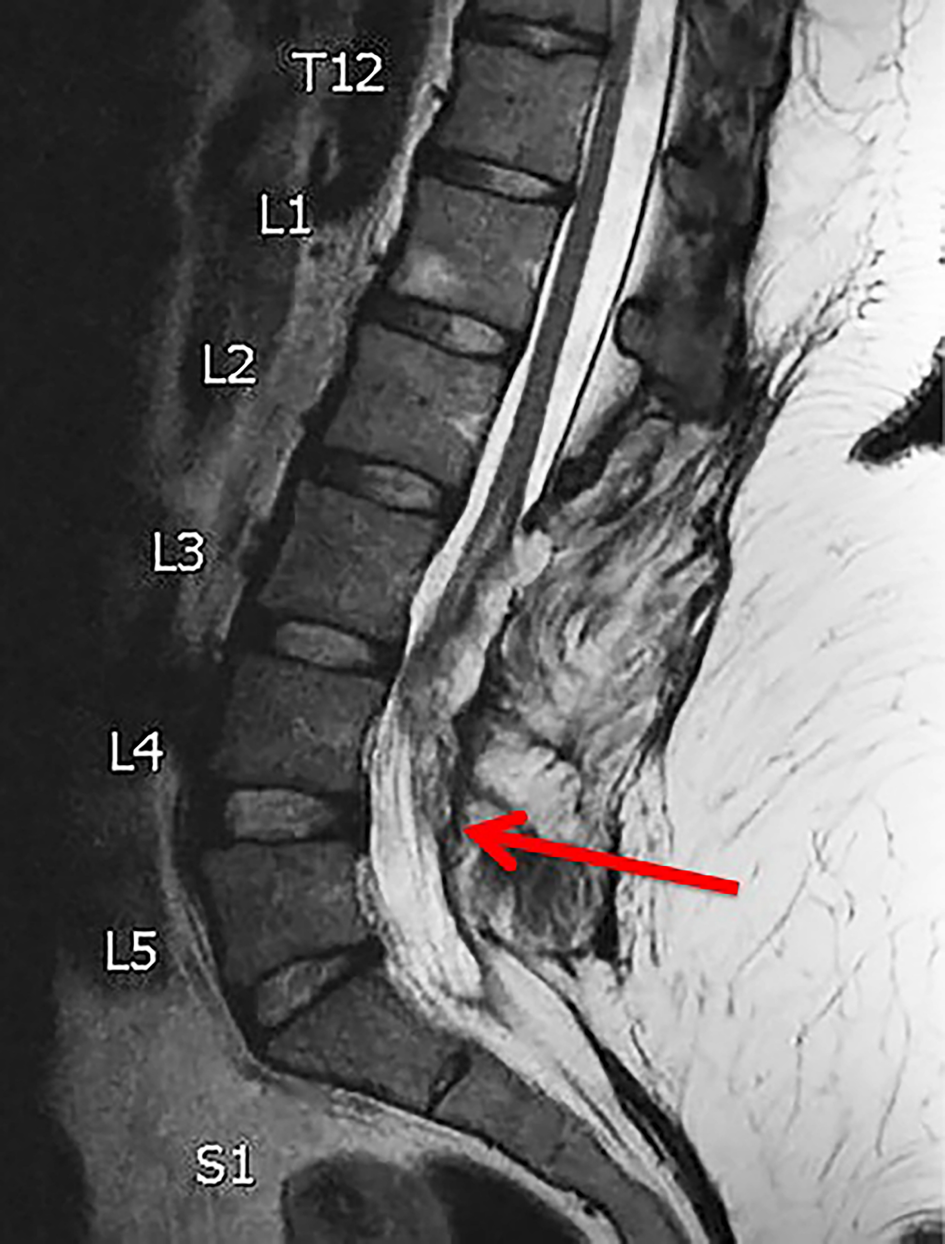
Preoperative MRI Sagittal T2 magnetic resonance imaging (MRI) demonstrating recurrent spinal cord tethering (demarcated by the red arrow)

A 21-year-old female, with a history of myelomeningocele repair as an infant, followed by two subsequent detethering procedures, presented with progressive lower extremity numbness and weakness in addition to worsening bladder dysfunction. She required straight catheterization for voiding. Her strength was 4/5 in the right lower extremity, 4-/5 in the left lower extremity, with 2/5 left dorsiflexion. She required the use of a walker for ambulation greater than one block. A magnetic resonance imaging (MRI) scan demonstrated tethered cord recurrence (Figure 1). Given her multiple previous detethering procedures (four in total), spinal shortening was offered to the patient to avoid the complication profile of redo detethering surgery.

Operative report

**Figure 2:**
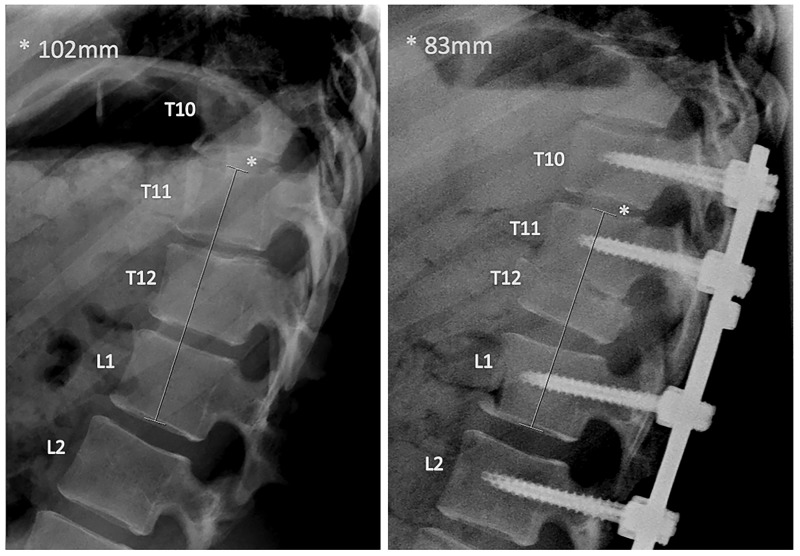
Pre- and Postoperative Lateral Radiograph (left and right, respectively) The preoperative thoracolumbar lateral radiograph on the left demonstrates the initial spinal column length in millimeters. The postoperative thoracolumbar lateral radiograph on the right demonstrates partial T12 corpectomy, with associated pedicle, lamina, and facet removal, including instrumentation. Nineteen millimeters of overall spinal column shortening was achieved.

The patient was positioned in the left lateral decubitus position with the use of neuromonitoring. Preoperative lateral thoracolumbar radiography demonstrated the initial spinal column length (Figure 2). The T11/12 vertebral bodies were outlined and the overlying rib was marked out as our planned incision, approximately five centimeters in length. A skin incision was made followed by soft tissue dissection and resection of the underlying rib. At this point, the pleural cavity was identified. Blunt dissection was carried out in the retropleural, retrodiaphragmatic space down to the lateral aspect of the T11/T12 vertebral bodies, which was confirmed with radiography. A self-retaining retractor system was placed, creating our working corridor.

A discectomy at T11/12 was completed, followed by partial corpectomy from the top of the T12 vertebral body down to the inferior aspect of the T12 pedicle. The ipsilateral pedicle was then removed. The patient was then turned to the prone position and a skin incision was made from T10-L2. Pedicle screws were placed at T10, T11, L1, and L2. A temporary rod was placed and laminectomy was completed at T12. This was followed by a partial laminectomy of T11 with facetectomy at T11/T12 to release the posterior elements in addition to the removal of the remaining T12 pedicle. Permanent rods were sequentially placed followed by the compression of the vertebral column. Neuromonitoring remained at baseline throughout the entirety of the procedure.

Postoperative course

**Figure 3:**
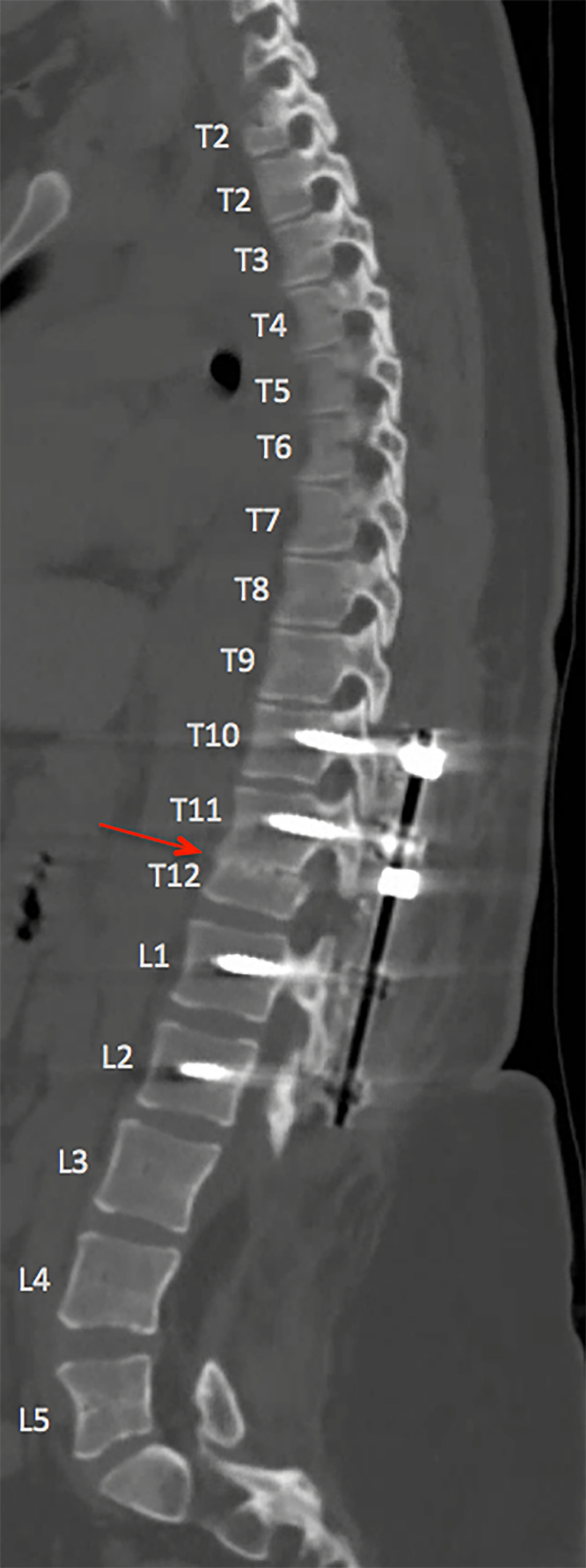
Six-Month Postoperative Sagittal CT Scan Postoperative sagittal computed tomography (CT) scan of the thoracolumbar spine at six months, demonstrating spinal shorting at T12 with posterior instrumented fusion from T10, T11, L1, and L2. Sagittal alignment is maintained with early fusion demonstrated at the vertebral osteotomy site (demarcated by the red arrow).

The patient remained at her neurologic baseline postoperatively and was discharged home on postoperative Day 9. Standing lateral radiographs demonstrated 19 millimeters (mm) of spinal column shortening as compared to preoperative radiographs (Figure 2). Her postoperative neurologic exam remained stable as compared to her preoperative exam. Follow-up thoracolumbar radiography demonstrated intact instrumentation shortening of T11/T12. At the six-month follow-up, the patient reported decreased lower extremity radicular pain and improved control of the bowel and bladder function although she still required straight catheterization. She could ambulate for much of her daily activities with only the use of an ankle and foot orthoses, an improvement from her normal use of her walker. A computed tomography (CT) scan of the lumbar/thoracic spine demonstrated preserved sagittal balance and early fusion formation at T11/12 (Figure 3).

## Discussion

Recurrent TCS remains a challenging entity to manage. Surgery for repeat detethering is associated with complication rates of 5%-50%, which may include CSF leaks, additional scarring that results in further tethering of the spinal cord, and direct iatrogenic neural injury [12-13]. SSVO is a novel surgical procedure that decreases tension on the spinal cord without direct manipulation of the neural elements. Small series demonstrate successful outcomes with a posterior approach to SSVO in recurrent tethered cord [12,17-18]. SSVO, as compared with direct redo-detethering achieves a reduction in spinal cord tension while avoiding direct neurologic injury, CSF leaks, and the formation of adhesions that may result in further tethering.

This case represents the first SSVO case via a lateral retropleural approach combined with a posterior fusion. The lateral approach allows for direct access to the anterior vertebral column and is considered a minimally invasive approach compared to posterior approaches to the anterior spinal column for corpectomy. Although we did complete an open posterior fusion in this case, we believe an entirely minimally invasive vertebral shortening procedure is feasible by utilizing a tubular retractor system to complete a posterior laminectomy, facetectomy, and pedicle resection, followed by a percutaneous instrumented fusion. The benefits of minimally invasive spinal surgery include decreased blood loss, shorter length of hospital stay, decreased soft tissue trauma, reduced postoperative pain, and earlier return to work [19-20].

## Conclusions

This case further supports SSVO as a treatment option for recurrent TCS while also demonstrating the potential for the lateral approach as well as minimally invasive approaches for spinal shortening procedures.
